# New Insights on the Zika Virus Arrival in the Americas and Spatiotemporal Reconstruction of the Epidemic Dynamics in Brazil

**DOI:** 10.3390/v13010012

**Published:** 2020-12-23

**Authors:** Larissa Catharina Costa, Rafael Valente Veiga, Juliane Fonseca Oliveira, Moreno S. Rodrigues, Roberto F. S. Andrade, Enny S. Paixão, Maria Glória Teixeira, Maria da Conceição N. Costa, Luciana L. Cardim, Eduardo H. Carmo, Wanderson K. Oliveira, José Í. K. Gonçalves, Qeren H. R. F. Fernandes, Maurício L. Barreto, Artur T. L. Queiroz, Tiago Gräf

**Affiliations:** 1 Center of Data and Knowledge Integration for Health (CIDACS), Gonçalo Moniz Institute, Oswaldo Cruz Foundation, Salvador 41745-715, Bahia, Brazil; rafaelvalenteveiga@gmail.com (R.V.V.); julianlanzin@gmail.com (J.F.O.); randrade@ufba.br (R.F.S.A.); enny.cruz@lshtm.ac.uk (E.S.P.); magloria@ufba.br (M.G.T.); mariacncosta@hotmail.com (M.d.C.N.C.); lucianacardim@yahoo.com.br (L.L.C.); ehcarmo@gmail.com (E.H.C.); irahe22@gmail.com (J.Í.K.G.); qerenferreira@gmail.com (Q.H.R.F.F.); mauricio@ufba.br (M.L.B.); artur.queiroz@fiocruz.br (A.T.L.Q.); 2 Oswaldo Cruz Foundation, Porto Velho 76812-245, Rondônia, Brazil; rodriguesmsb@gmail.com; 3 Physics Institute, Federal University of Bahia, Salvador 40210-340, Bahia, Brazil; 4 Department of Infectious Disease Epidemiology, London School of Hygiene and Tropical Medicine, London WC1E 7HT, UK; 5 Collective Health Institute, Federal University of Bahia, Salvador 40110-040, Bahia, Brazil; 6 Ministry of Defense Hospital das Armed Forces, Technical Directorate of Education and Research, Brasilia 70675-731, Federal District, Brazil; wkoliveira@gmail.com; 7 Gonçalo Moniz Institute, Oswaldo Cruz Foundation, Salvador 40296-710, Bahia, Brazil; tiago.graf@fiocruz.br

**Keywords:** ZIKV, Brazil, phylogeography, molecular clock

## Abstract

Zika virus (ZIKV) became a worldwide public health emergency after its introduction in the Americas. Brazil was implicated as central in the ZIKV dispersion, however, a better understanding of the pathways the virus took to arrive in Brazil and the dispersion within the country is needed. An updated genome dataset was assembled with publicly available data. Bayesian phylogeography methods were applied to reconstruct the spatiotemporal history of ZIKV in the Americas and with more detail inside Brazil. Our analyses reconstructed the Brazilian state of Pernambuco as the likely point of introduction of ZIKV in Brazil, possibly during the 2013 Confederations Cup. Pernambuco played an important role in spreading the virus to other Brazilian states. Our results also underscore the long cryptic circulation of ZIKV in all analyzed locations in Brazil. Conclusions: This study brings new insights about the early moments of ZIKV in the Americas, especially regarding the Brazil-Haiti cluster at the base of the American clade and describing for the first time migration patterns within Brazil.

## 1. Introduction

Zika virus (ZIKV) is a positive-stranded RNA arbovirus belonging to the *Flaviviridae* family and *Flavivirus* genus. It was first isolated in 1947 from the serum of a febrile Rhesus monkey during a yellow fever study in a forest named “Zika’’ near Lake Victoria in Uganda [1]. In humans, ZIKV was first isolated in Nigeria in 1954 [2]. For 60 years, ZIKV was confined to an equatorial zone across Africa and Asia causing sporadic infections in humans. In 2007, the outbreak of the virus in Yap Island, Federated States of Micronesia, marked the first detection of ZIKV in Oceania [3]. Later on, in 2013–2014, the virus spread to French Polynesia [4] and other Pacific Islands, such as Cook Islands [5], Easter Island [6], New Caledonia [7], and Tahiti [8].

Phylogenetic reconstruction of ZIKV evolutionary history shows that the virus emerged in the early 1900s in Uganda, and dispersed through Western Africa and Asia (1930–1950) when it diverged into two genetic lineages: the African and Asian lineages [9,10,11]. Studies analyzing ZIKV genome sequences have revealed that the virus was introduced in the American continent through Brazil between late 2013 and early 2014 and the most likely origin was French Polynesia [9,12]. Another approach to applying mathematical modeling had similar results. The authors estimated the expected amount of infected individuals during a travel flux between Brazil and French Polynesia, concluding that ZIKV might have been introduced and established in Brazil between October 2013 and March 2014 [13]. However, it was only in April 2015 that autochthonous cases of ZIKV infection were laboratory-confirmed in Brazil [14,15]. From Brazil it spread to other South and Central American and Caribbean countries in 2015 [16,17,18] and later reached the USA, in 2016, most likely coming from the Caribbean [19].

Although several studies have investigated the evolutionary history of ZIKV Asian lineage, with a special focus on the dispersion patterns across the American continent, little is known about the virus diffusion within Brazil, the epicenter of the worldwide cluster of microcephaly cases and other neurological disorders related to ZIKV congenital transmission [20,21]. In this work, we present a comprehensive study based on an updated set of full-length and near-complete ZIKV genomes from the Caribbean, Central, North, and South Americas, with a special focus on Brazil. By applying methods on molecular clock and phylogeography, we discuss the emergence of ZIKV in the Americas and bring new insights about the early spread in Brazil and the role of Pernambuco State as a starting point and dissemination hub. 

## 2. Materials and Methods

### 2.1. Data Collection

**Figure 1:**
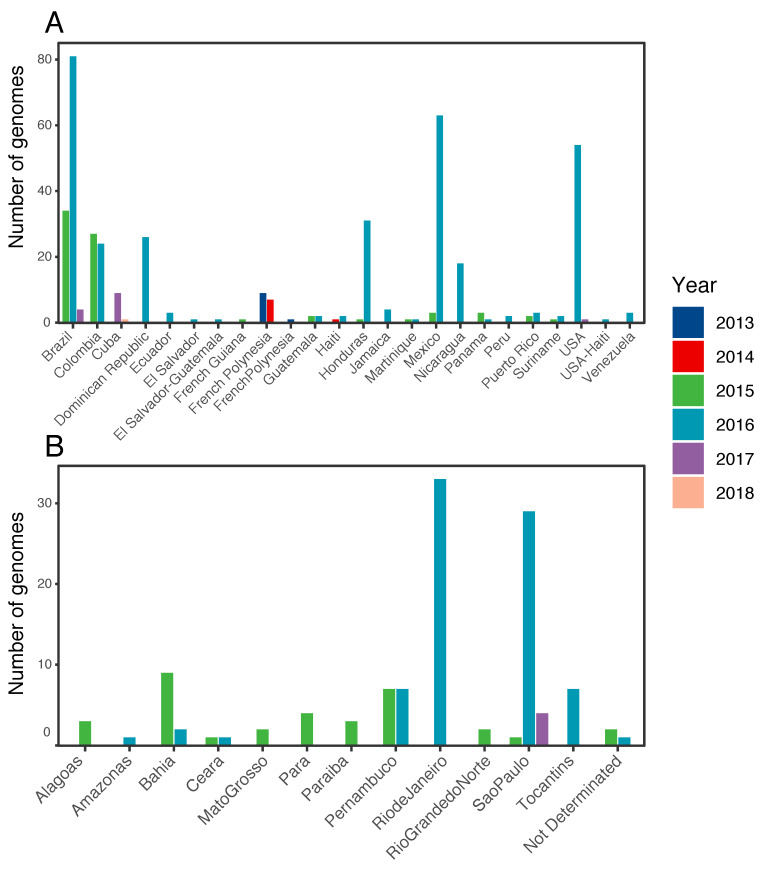
Place and year of sampling of the Zika virus (ZIKV) genomes analyzed in this study. (**A**) American countries and French Polynesia. (**B**) Brazilian states by year before the down sampling.

All complete or near-complete ZIKV genomes from American countries and French Polynesia available in GenBank up to January 2019 were retrieved (*n* = 493). Sequences without a sampling date and with more than 50% of uninformative positions (N) after alignment were removed, resulting in a dataset of 431 genomes (Figure 1A and Table S1). The database from Brazil consisted of 119 genome sequences spanning 12 states in four regions of the country (Midwest, North, Southwest, and Northwest) (Figure 1B and Table S2).

The notification of suspected cases of acute ZIKV infection is performed through the Sistema de Informação de Agravos de Notificação (Information System for Notifiable Diseases, SINAN) [22]. SINAN/Zika database was used to obtain registered cases of Zika in Brazilian states from 2015–2017. Notifications before April 2015 were excluded, because ZIKV was only confirmed in Brazil in April 2015 [14]. Therefore, ZIKV records in SINAN could be annotation errors or subsequently reclassified cases. Nevertheless, there were few reports in this period and their removal is unlikely to impact our analysis.

### 2.2. Phylogenetic and Phylogeographic Analyses

Sequence alignment was performed using MAFFT [23] and visually inspected in Aliview [24]. A phylogenetic tree was inferred in a maximum-likelihood analysis (ML) using IQ-Tree [25]. The best-fitted substitution model was tested with the ModelFinder [26] as implemented in the IQ-Tree. Trees were viewed in FigTree and the temporal signal of this phylogeny was assessed in Tempest [27].

Time-scaled phylogenetic trees were inferred using the BEAST/BEAGLE package [28]. The analysis was set with the SRD06 site model [29], the uncorrelated lognormal distributed relaxed clock model [30], and the SkyGrid coalescent model [31]. A discrete phylogeographical model [32] was used to reconstruct the spatial diffusion of the virus across the compiled dataset sampling locations. Phylogeographic analyses were then performed by applying an asymmetric model of location transitioning, coupled with the Bayesian stochastic search variable selection (BSSVS) procedure. We complemented this analysis with a Markov jump estimation. 

In order to understand with greater detail, the ZIKV dispersion routes inside Brazil, we created a subset of ZIKV genomes sampled only in Brazil. This procedure was possible since our worldwide dataset showed a single introduction event in Brazil. Discrete locations were then set as the state of sampling and to diminish the impact of sampling heterogeneity in the phylogeography reconstruction, down sampling was performed in the two states (São Paulo and Rio de Janeiro) with the highest number of genomes. This procedure was performed randomly three times, generating three datasets that were analyzed independently.

Monte Carlo Markov chains (MCMC) were run sufficiently long to ensure stationarity of the chains and good ESS values, as diagnosed in Tracer software [33]. The maximum clade credibility (MCC) tree was obtained with the TreeAnnotator software and visualized in FigTree v1.4.2. Viral transition rates between geographical locations were summarized using SPREAD3 [34] where well-supported transitions were identified by the Bayes Factor (BF) support higher than 3. 

The CIPRES Science gateway platform [35] was used to run MAFFT, IQ-tree, and BEAST.

## 3. Results

### 3.1. ZIKV in the Americas

**Figure 2:**
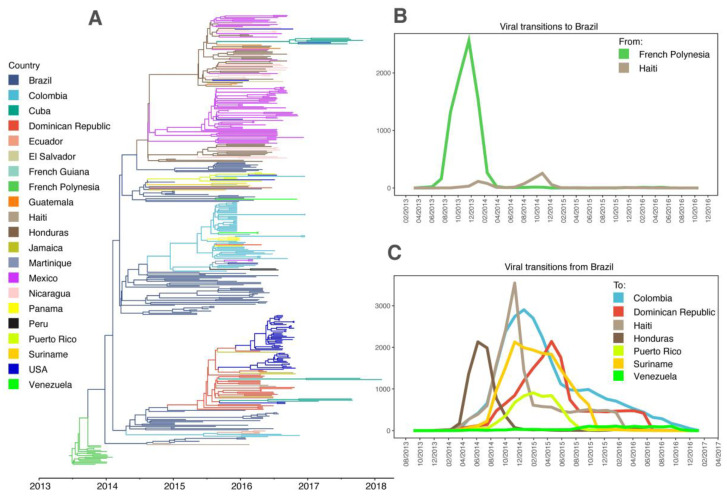
Spatial and temporal phylogenetic analyses of ZIKV in Americas. (**A**) Maximum clade credibility (MCC) phylogeny ZIKV genomes showing introduction in the Americas (Central, North, and South) and the Caribbean. (**B**) Supported (BF > 3.0) viral transitions through time of ZIKV lineages with destination to Brazil according to Markov Jumps analysis; (**C**) Supported (BF > 3.0) viral transitions through time of ZIKV lineages from Brazil to other countries.

In the view of constant new genome data release, we firstly performed an analysis of the full ZIKV diversity in the Americas. Our updated dataset contains 431 genomes from the Caribbean, Central, North, and South American countries, besides French Polynesia, dated from 2013 to 2018 (Figure 1A). In line with previous studies, our results estimated that ZIKV was introduced in Brazil from French Polynesia and the time of the most recent common ancestor (tMRCA) of all the American clade was estimated between November 2013 and March 2014 (95% HPD (height posterior density)) (Figure 2). Viral transitions from French Polynesia to Brazil were highly supported (BF = 658.44) and were mainly concentrated in late 2013, although they started as early as August 2013, highlighting the uncertainties around the moment of introduction (Figure 2B). Besides French Polynesia, the only country that was estimated to have seeded ZIKV lineages into Brazil was Haiti (BF = 3.1). Genomes from both countries grouped together in a small sibling clade (pp = 0.82) to the whole American ZIKV clade and seemed to have exchanged viral lineages around the middle of 2014. However, this viral transition was asymmetric since Brazil was estimated to have contributed much more intensively to the Haitian epidemic than the opposite way (Figure 2A,B). Moreover, Brazil is highly supported (pp = 0.97) as the state node of the MRCA of the whole American clade and of the small sibling clade (pp = 0.93). From Brazil, ZIKV rapidly disseminated to other countries, starting from Haiti in the middle of 2014, then, from late 2014 onwards to Colombia, Dominican Republic, Honduras, Puerto Rico, Suriname, and Venezuela (Figure 2C). Supplementary Table S3 summarizes the supported transitions from Brazil to other American countries.

### 3.2. The ZIKV Dissemination across the Brazilian States

To understand the propagation of Zika inside Brazil, 119 genome sequences from 12 states from Central-West, North, Northeast, and Southeast regions were used (Figure 1B). Aiming to diminish the impact of sampling heterogeneity in the phylogeographical reconstruction, sequences from Rio de Janeiro (*n* = 33) and São Paulo (*n* = 34) were randomly down sampled to generate three datasets with a more balanced distribution of samples per state. The three datasets were analyzed separately and showed very similar results; therefore, here we describe the results of the first down sampled dataset (DS1).

**Figure 3:**
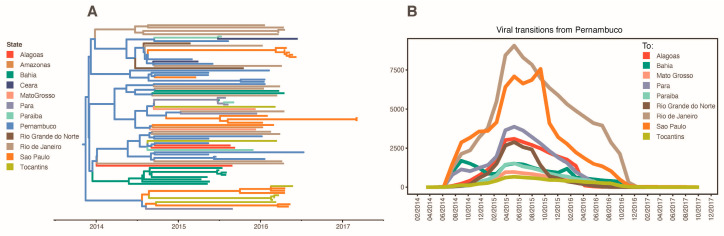
Spatiotemporal reconstruction of ZIKV in Brazil. (**A**) MCC tree of ZIKV genomes isolated in Brazil. Branches are colored according to the state reconstructed in the phylogeographic analysis. (**B**) Supported (BF > 3.0) ZIKV lineages transitions, as calculated by Markov’s jumps, from Pernambuco to other Brazilian states.

Our analysis estimates that ZIKV has arrived in Brazil through Pernambuco State (pp = 0.94) and from there it spread to other states (Figure 3A). It is possible to observe that Pernambuco was responsible for introducing ZIKV in at least nine Brazilian states between the middle of 2014 and late 2016, with the bulk of transitions happening around the middle of 2015 (Figure 3B). Supplementary Table S4 summarizes all supported transitions from Pernambuco to other Brazilian states.

**Figure 4:**
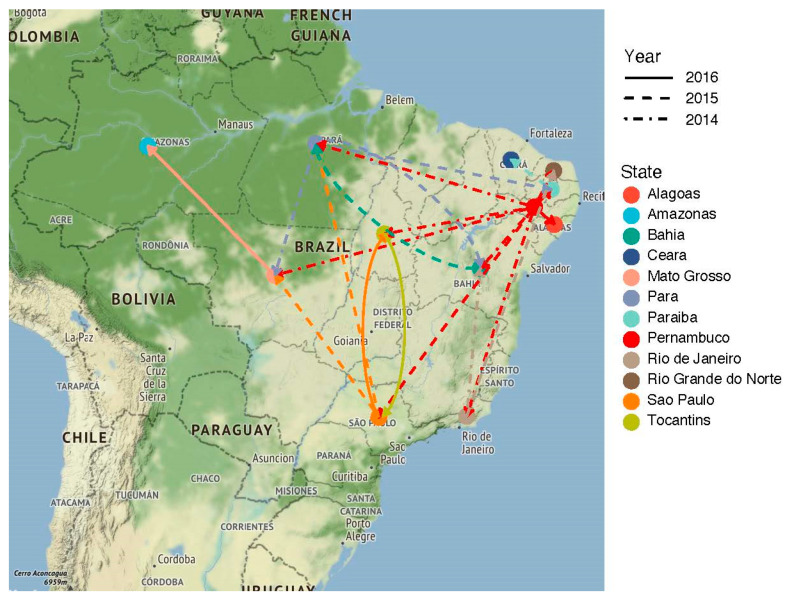
ZIKV migration patterns between 2014–2016 within Brazil.

**Figure 5:**
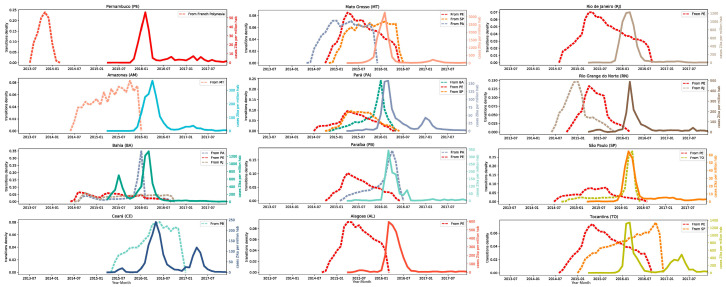
ZIKV **transition** dynamics between Brazilian states and epidemic history. Each panel shows the number of viral transitions through time, as estimated by Markov Jumps analysis, (dashed lines) colored according to the state of origin and the number of confirmed cases per million habitants (solid line).

Although Pernambuco was estimated to be the central hub of ZIKV spread during the cryptic period of virus circulation (before first cases were identified in Brazil and a diagnostic algorithm was developed), other states also have a role in the spreading process in the following seasons (Figure 4 and Figure 5). Interesting to observe, São Paulo was responsible for the introduction of virus lineages in the inland states of Mato Grosso in 2015 and Tocantins in 2016, aside from the northern state of Pará in 2015. The only two states present in our dataset not seeded by Pernambuco were Amazonas and Ceará, which had ZIKV lineages introduced from Mato Grosso in 2015 and Paraíba in 2016, respectively. The states of Pará and Bahia received a large diversity of lineages, with origin in three different states. 

Finally, we compared the epidemic dynamics of each state sampled in our dataset, with the ZIKV lineages transitions through time (Figure 5). By doing this, it becomes clear that the virus has circulated for a long period without being noticed. This cryptic circulation might be related to the high number of asymptomatic individuals and the difficulties in distinguishing among other arboviruses, like dengue or chikungunya [36]. Most likely, after the arrival of the virus in each state, the rate of infection was too low to attract the attention of the epidemiological surveillance agencies to this new disease.

Although the first ZIKV cases started to be reported at the beginning of 2015, most states only have a noticeable epidemic season in 2016, while our analyses have estimated that the virus was in circulation at least for a year before that. The first notification of ZIKV occurred in Bahia state [14] and it was the only one to report a significant ZIKV peak in 2015, reaching around 700 cases per million inhabitants [37]. Unlike Pernambuco, the most densely populated area in Bahia has high rainfall in this period enabling the proliferation of the disease vector.

## 4. Discussion

In this study, we aimed to review the diffusion patterns of ZIKV in the American continent and to understand in detail the dispersion of ZIKV inside Brazil. In agreement with previous studies [9,11,12], our results found that ZIKV arrived in the Americas through Brazil and from there it spread to other countries. Although our analysis reconstructed some viral lineage exchanges between Brazil and Haiti during the cryptic epidemic period, we did not find evidence to support that Haiti was the entrance point of ZIKV in the Americas, as recently suggested [38]. Indeed, Brazil was estimated as the ancestral state of the small Brazil-Haiti clade, even though the basal sequences were from Haiti and the oldest have been isolated to December 2014 (GenBank accession number KU509998). Curiously, this is the first ZIKV genome isolated in the Americas, however, due to the huge gap of samples between the estimated introductory time in late 2013 and the first detected by a laboratory in early 2015, [14,15] only robust phylogeographic reconstruction can reveal the history of ZIKV dispersion during the cryptic phase.

The entrance date of ZIKV in Brazil, and consequently in the Americas, is still debatable. Initially, the virus entry was related to the FIFA World Cup (June 2014), however, the Pacific Islands that reported the ZIKV outbreak, whose genetic diversity is the closest to the American lineage, were not competing and the period that this competition took place is not compatible with the tMRCA of the American clade (November 2013 to March 2014, 95% HPD). Another hypothesis of virus entry is the Va’a World Sprint Championship canoe race, hosted by Brazil in August 2014 with teams from New Caledonia and the Cook Islands competing. However, again the time frame of this sport event and molecular clock estimates are not in agreement. Lastly, the 2013 Confederations Cup soccer tournament (June 2013), happened in Brazil, and Tahiti, the largest island in French Polynesia, was competing. Although this event seems to have taken place too early in comparison to the estimated tMRCA, the Markov jumps analysis performed here supports transitions happening as early as August 2013, which is consistent with the size of the branch connecting the American clade to the French Polynesia genomes (Figure 2A). Besides that, due to the lack of genomes from 2014, the estimated tMRCA can be biased towards the present. Another issue with the 2013 Confederations Cup hypothesis is that the French Polynesia outbreak was detected in October 2013 [39], which predates the event by some months, however, as observed in Brazil, ZIKV might have been cryptically circulating in French Polynesia much before the first detected cases. 

In Brazil, as already mentioned, little is known about the propagation of the virus inside the country [40], and here, this study, for the first time, performed a phylogeographic reconstruction in a resolution of country states. Our results revealed that the Pernambuco State was likely the introduction point of ZIKV in Brazil and played an important role in the dissemination to other Brazilian states. In agreement with the 2013 Confederations Cup hypothesis, the team from Tahiti played in Recife, the capital of Pernambuco. Important to note that Tahiti also played in the southeastern cities Belo Horizonte and Rio de Janeiro in June 2013. However, in this period of the year, the ﻿mosquito-borne viral suitability measure, also called the index P [38], drops substantially for Belo Horizonte and Rio de Janeiro, while in Recife climate conditions are permissive for arbovirus transmission all year long. Therefore, it is possible that an effective introduction of ZIKV happened in Pernambuco during the 2013 Confederations Cup, seeding the viral lineage that circulated cryptically for more than a year before the emergence of the first recorded cases. In addition, the state of Pernambuco is a tourist spot, receiving national and international tourism as well as business events all year long. Moreover, the 2014 World Cup also took place in Pernambuco, which might have foster viral transitions to other states and countries. 

It is worth mentioning that the emergence of an aggregate of cases of unknown exanthematic disease occurred in cities of the states of Pernambuco (PE), Rio Grande do Norte (RN), and Bahia (BA), all situated in the Northeast region of Brazil, in late 2014 [41], but only in April 2015 was it identified as ZIKV [14,15]. Therefore, our findings are in line with both the surveillance information system and with the analyses of viral genomes firstly presented by Farias et al. [12].

In summary, our findings provide further evidence that ZIKV arrived in the Americas through Brazil, coming from French Polynesia, likely during the 2013 Confederations Cup. Our study, for the first time, investigates ZIKV spread within Brazilian states, revealing that Pernambuco State was likely the entrance point and hub of dispersion to other Brazilian locations. It has so far been the most detailed description of the spread of the virus within Brazil, which has become the epicenter of a Public Health Emergency of International Concern due to the microcephaly epidemic caused by congenital transmission ZIKV, an unknown condition until 2015 [21]. Historical time events (i.e., Confederations Cup) and seasonal variation in arboviral suitability estimations support Pernambuco as the origin of the Brazilian ZIKV epidemic. In conclusion, this study highlights the potential of genomic epidemiology to answer crucial public health questions. These results highlight that technical and methodological advances in the field of genomic studies must be applied to viral surveillance. Phylogenetics has the potential to elucidate the origin, mutations impact, and path of infectious agents, especially those emerging. Such information can be useful to support the development of prediction models and the definition of possible control strategies.

## Supplementary Materials

The following are available online at https://www.mdpi.com/1999-4915/13/1/12/s1, Table S1: Full-length ZIKV genomes and near-complete ZIKV genomes from Central, North and South Americas and French Polynesia (>5000 nucleotides) available in GenBank up to January 2019. Table S2: Full-length ZIKV genomes and near-complete ZIKV genomes from Brazil (>5000 nucleotides) available in GenBank up to January 2019. Table S3: Median and 95% HPD of estimated introductions, as calculated by MCCTree, from Brazil to other American countries. Table S4: Median and 95% HPD of estimated introductions, as calculated by MCC Tree, from Pernambuco to other Brazilian states.
