# Supplementary material for: New Insights on the Zika Virus Arrival in the Americas and Spatiotemporal Reconstruction of the Epidemic Dynamics in Brazil
Source: Viruses. 2020 Dec 23;13(1):12. doi: 10.3390/v13010012 (PMC7824532; doi:10.3390/v13010012)
Supplement: Supplementary file 1 [file viruses-13-00012-s001.zip › Supplementary_Table_S1.docx]

Supplementary Table S1: Full-length ZIKV genomes and near-complete ZIKV genomes from Central, North and South Americas and French Polynesia (> 5000 nucleotides) available in GenBank up to January, 2019.

| **Accession Number** | **Country** | **Collection Date** |
| --- | --- | --- |
| KJ776791 | FrenchPolynesia | 2013-11-28 |
| KU312312 | Suriname | 2015-10-02 |
| KU321639 | Brazil | 2015-3 |
| KU365777 | Brazil | 2015 |
| KU365778 | Brazil | 2015 |
| KU365779 | Brazil | 2015 |
| KU365780 | Brazil | 2015 |
| KU497555 | Brazil | 2015-11-30 |
| KU501215 | Puerto Rico | 2015-12-01 |
| KU501216 | Guatemala | 2015-12-01 |
| KU501217 | Guatemala | 2015-11-01 |
| KU509998 | Haiti | 2014-12-12 |
| KU647676 | Martinique | 2015-12 |
| KU707826 | Brazil | 2015-07-01 |
| KU729217 | Brazil | 2015 |
| KU729218 | Brazil | 2015 |
| KU758877 | French Guiana | 2015-12 |
| KU820897 | Colombia | 2015-12 |
| KU870645 | USA | 2016-02-02 |
| KU922923 | Mexico | 2016-02-25 |
| KU922960 | Mexico | 2016-02-25 |
| KU926309 | Brazil | 2016-01-14 |
| KU926310 | Brazil | 2016-01-29 |
| KU937936 | Suriname | 2016-02-11 |
| KU940224 | Brazil | 2015-08-01 |
| KU940227 | Brazil | 2015-07-15 |
| KU940228 | Brazil | 2015-07-01 |
| KX051563 | USA-Haiti | 2016-02-05 |
| KX101060 | Brazil | 2015-5 |
| KX101061 | Brazil | 2015-5 |
| KX101064 | Brazil | 2015-4 |
| KX101066 | Brazil | 2015-5 |
| KX156774 | Panama | 2015-12-18 |
| KX156775 | Panama | 2015-12-11 |
| KX156776 | Panama | 2015-12-18 |
| KX197192 | Brazil | 2015 |
| KX198135 | Panama | 2016 |
| KX247632 | Mexico | 2015-11 |
| KX247646 | Colombia | 2016-02-09 |
| KX262887 | Honduras | 2016-01-06 |
| KX280026 | Brazil | 2015 |
| KX369547 | French Polynesia | 2013-10-25 |
| KX377337 | Puerto Rico | 2015-12 |
| KX421194 | Nicaragua | 2016-01-13 |
| KX421195 | Nicaragua | 2016-01-19 |
| KX446950 | Mexico | 2016-01-01 |
| KX446951 | Mexico | 2016-01-01 |
| KX447509 | French Polynesia | 2013-12 |
| KX447510 | French Polynesia | 2013-12 |
| KX447511 | French Polynesia | 2014-1 |
| KX447512 | French Polynesia | 2013-12 |
| KX447513 | French Polynesia | 2013-12 |
| KX447514 | French Polynesia | 2014-1 |
| KX447515 | French Polynesia | 2013-11 |
| KX447516 | French Polynesia | 2014-1 |
| KX447517 | French Polynesia | 2014-1 |
| KX447518 | French Polynesia | 2013-12 |
| KX447519 | French Polynesia | 2013-11 |
| KX447520 | French Polynesia | 2014-1 |
| KX447521 | French Polynesia | 2014-2 |
| KX520666 | Brazil | 2015-8 |
| KX548902 | Colombia | 2015-10-07 |
| KX694534 | Honduras | 2015-01-06 |
| KX702400 | Venezuela | 2016-03-25 |
| KX766028 | Dominican Republic | 2016-06-06 |
| KX811222 | Brazil | 2016-06-14 |
| KX827268 | USA | 2016-01-01 |
| KX830930 | Brazil | 2016-03-01 |
| KX832731 | USA | 2016-08-24 |
| KX838904 | USA | 2016-08-22 |
| KX856011 | Mexico | 2016-01-01 |
| KX879603 | Ecuador | 2016-4 |
| KX879604 | Ecuador | 2016-4 |
| KX893855 | Venezuela | 2016-03-25 |
| KX906952 | Honduras | 2016-04-16 |
| KX922703 | USA | 2016-07-19 |
| KX922705 | USA | 2016-08-05 |
| KX922706 | USA | 2016-08-05 |
| KX922707 | USA | 2016-08-17 |
| KX922708 | USA | 2016-09-04 |
| KX986760 | Brazil | 2016-2 |
| KY014295 | USA | 2016-06-22 |
| KY014296 | Brazil | 2016-04-18 |
| KY014297 | Brazil | 2016-04-12 |
| KY014298 | USA | 2016-08-05 |
| KY014299 | USA | 2016-09-04 |
| KY014300 | Dominican Republic | 2016-04-20 |
| KY014301 | Brazil | 2016-04-13 |
| KY014302 | Dominican Republic | 2016-04-21 |
| KY014303 | Dominican Republic | 2016-04-11 |
| KY014304 | Dominican Republic | 2016-04-18 |
| KY014305 | Dominican Republic | 2016-04-05 |
| KY014306 | Honduras | 2016-06-10 |
| KY014307 | Brazil | 2016-03-28 |
| KY014308 | Brazil | 2016-03-23 |
| KY014309 | Brazil | 2016-03-28 |
| KY014310 | Honduras | 2016-05-09 |
| KY014311 | Honduras | 2016-05-12 |
| KY014312 | Honduras | 2016-05-13 |
| KY014313 | Brazil | 2016-04-05 |
| KY014314 | Dominican Republic | 2016-06-14 |
| KY014315 | Honduras | 2016-06-06 |
| KY014316 | USA | 2016-08-17 |
| KY014317 | Brazil | 2016-03-21 |
| KY014318 | Dominican Republic | 2016-04-27 |
| KY014319 | Honduras | 2016-04-30 |
| KY014320 | Brazil | 2016-03-23 |
| KY014321 | Dominican Republic | 2016-04-11 |
| KY014322 | USA | 2016-08-23 |
| KY014323 | USA | 2016-08-23 |
| KY014324 | USA | 2016-08-22 |
| KY014325 | USA | 2016-08-02 |
| KY014326 | USA | 2016-08-05 |
| KY014327 | Honduras | 2016-06-09 |
| KY075932 | USA | 2016-03-22 |
| KY075933 | USA | 2016-06-21 |
| KY075934 | USA | 2016-07-03 |
| KY075935 | USA | 2016-07-16 |
| KY075936 | USA | 2016-08-04 |
| KY075937 | USA | 2016-09-09 |
| KY075938 | USA | 2016-09-20 |
| KY075939 | USA | 2016-10-05 |
| KY120348 | Mexico | 2016-03-03 |
| KY120349 | Mexico | 2016-03-03 |
| KY272991 | Brazil | 2016-02-12 |
| KY317936 | Colombia | 2016-01-16 |
| KY317937 | Colombia | 2016-01-07 |
| KY317938 | Colombia | 2016-01-10 |
| KY317939 | Colombia | 2016-01-06 |
| KY317940 | Colombia | 2016-01-09 |
| KY325464 | USA | 2016-09-19 |
| KY325466 | USA | 2016-07-28 |
| KY325467 | USA | 2016-07-29 |
| KY325468 | USA | 2016-08-04 |
| KY325469 | USA | 2016-08-04 |
| KY325470 | USA | 2016-08-04 |
| KY325471 | USA | 2016-08-08 |
| KY325473 | USA | 2016-08-31 |
| KY325474 | USA | 2016-09-13 |
| KY325475 | USA | 2016-10-05 |
| KY325476 | USA | 2016-10-11 |
| KY325477 | USA | 2016-10-03 |
| KY325478 | USA | 2016-09-26 |
| KY325479 | USA | 2016-09-28 |
| KY325480 | USA | 2016-07-28 |
| KY325481 | USA | 2016-07-29 |
| KY325482 | USA | 2016-08-24 |
| KY325483 | USA | 2016-08-24 |
| KY328289 | Honduras | 2016-5 |
| KY348640 | Suriname | 2016-01-22 |
| KY441401 | Brazil | 2016-02-29 |
| KY441402 | Brazil | 2016-04-05 |
| KY441403 | Brazil | 2016-01-11 |
| KY558989 | Brazil | 2015-02-23 |
| KY558990 | Brazil | 2016-01-15 |
| KY558991 | Brazil | 2016-01-19 |
| KY558992 | Brazil | 2016-01-06 |
| KY558993 | Brazil | 2016-01-18 |
| KY558994 | Brazil | 2016-01-18 |
| KY558995 | Brazil | 2015-05-13 |
| KY558996 | Brazil | 2015-05-13 |
| KY558997 | Brazil | 2015-05-14 |
| KY558998 | Brazil | 2015-06-15 |
| KY558999 | Brazil | 2016-07-10 |
| KY559000 | Brazil | 2015-08-09 |
| KY559001 | Brazil | 2015-08-20 |
| KY559002 | Brazil | 2015-09-09 |
| KY559003 | Brazil | 2015-08-28 |
| KY559004 | Brazil | 2016-04-16 |
| KY559005 | Brazil | 2016-04-18 |
| KY559006 | Brazil | 2016-04-18 |
| KY559007 | Brazil | 2016-04-18 |
| KY559008 | Brazil | 2016-04-19 |
| KY559009 | Brazil | 2016-04-19 |
| KY559010 | Brazil | 2016-04-19 |
| KY559011 | Brazil | 2016-04-19 |
| KY559012 | Brazil | 2016-04-19 |
| KY559013 | Brazil | 2016-04-24 |
| KY559014 | Brazil | 2016-04-24 |
| KY559015 | Brazil | 2016-04-24 |
| KY559016 | Brazil | 2016-04-25 |
| KY559017 | Brazil | 2016-05-19 |
| KY559018 | Brazil | 2016-02-25 |
| KY559019 | Brazil | 2016-05-24 |
| KY559020 | Brazil | 2016-03-07 |
| KY559021 | Brazil | 2016-03-10 |
| KY559022 | Brazil | 2016-03-13 |
| KY559023 | Brazil | 2016-03-22 |
| KY559024 | Brazil | 2016-03-03 |
| KY559025 | Brazil | 2016-01-15 |
| KY559026 | Brazil | 2016-01-15 |
| KY559027 | Brazil | 2016-02-16 |
| KY559029 | Brazil | 2016-1 |
| KY559030 | Brazil | 2016-1 |
| KY559031 | Brazil | 2016-1 |
| KY559032 | Brazil | 2016-1 |
| KY606271 | Mexico | 2016-03-05 |
| KY606272 | Mexico | 2016-08-05 |
| KY606273 | Mexico | 2016-06-30 |
| KY606274 | Mexico | 2016-07-07 |
| KY631492 | Brazil | 2016-01-08 |
| KY631493 | Mexico | 2015-10-15 |
| KY631494 | Mexico | 2015-10-15 |
| KY648934 | Mexico | 2016 |
| KY693676 | Honduras | 2016-08-26 |
| KY693677 | Honduras | 2016-08-26 |
| KY693678 | Peru | 2016-06-28 |
| KY693679 | Peru | 2016-07-11 |
| KY693680 | Venezuela | 2016-10-19 |
| KY765318 | Nicaragua | 2016-07-10 |
| KY765319 | Nicaragua | 2016-05-29 |
| KY765320 | Nicaragua | 2016-05-29 |
| KY765322 | Nicaragua | 2016-06-29 |
| KY765323 | Nicaragua | 2016-06-30 |
| KY765324 | Nicaragua | 2016-07-07 |
| KY765325 | Nicaragua | 2016-04-22 |
| KY765326 | Nicaragua | 2016-06-30 |
| KY785410 | Brazil | 2016-03-27 |
| KY785412 | USA | 2016-07-22 |
| KY785413 | Dominican Republic | 2016-10-06 |
| KY785414 | Honduras | 2016-05-28 |
| KY785415 | Dominican Republic | 2016-04-11 |
| KY785416 | Honduras | 2016-04-28 |
| KY785417 | Colombia | 2016-04-06 |
| KY785418 | Honduras | 2016-05-13 |
| KY785419 | Jamaica | 2016-06-13 |
| KY785420 | Dominican Republic | 2016-04-18 |
| KY785422 | USA | 2016-09-09 |
| KY785423 | Dominican Republic | 2016-04-05 |
| KY785424 | Jamaica | 2016-07-10 |
| KY785425 | Dominican Republic | 2016-04-04 |
| KY785426 | Brazil | 2016-04-04 |
| KY785427 | Brazil | 2016-03-30 |
| KY785429 | Brazil | 2016-04-14 |
| KY785430 | Jamaica | 2016-09-28 |
| KY785431 | Honduras | 2016-06-13 |
| KY785432 | Jamaica | 2016-10-12 |
| KY785433 | Brazil | 2016-04-08 |
| KY785435 | Dominican Republic | 2016-07-07 |
| KY785437 | Brazil | 2016-03-14 |
| KY785439 | Brazil | 2016-04-08 |
| KY785441 | Dominican Republic | 2016-06-13 |
| KY785442 | Honduras | 2016-06-04 |
| KY785443 | USA | 2016-08-01 |
| KY785445 | USA | 2016-07-19 |
| KY785446 | Brazil | 2016-03-22 |
| KY785447 | Dominican Republic | 2016-09-22 |
| KY785448 | Honduras | 2016-06-10 |
| KY785449 | Dominican Republic | 2016-04-05 |
| KY785450 | Brazil | 2016-04-12 |
| KY785451 | Martinique | 2016-03-22 |
| KY785452 | Honduras | 2016-06-07 |
| KY785453 | Dominican Republic | 2016-05-10 |
| KY785454 | El Salvador-Guatemala | 2016-08-05 |
| KY785455 | Brazil | 2016-04-06 |
| KY785456 | Brazil | 2016-04-15 |
| KY785457 | USA | 2016-08-01 |
| KY785458 | Honduras | 2016-06-10 |
| KY785459 | USA | 2016-08-15 |
| KY785461 | Honduras | 2016-05-31 |
| KY785462 | Puerto Rico | 2016-06-26 |
| KY785463 | Dominican Republic | 2016-04-05 |
| KY785464 | Puerto Rico | 2016-04-13 |
| KY785465 | Dominican Republic | 2016-04-07 |
| KY785466 | Colombia | 2016-04-28 |
| KY785468 | USA | 2016-10-05 |
| KY785469 | Colombia | 2016-04-05 |
| KY785470 | Dominican Republic | 2016-06-18 |
| KY785471 | Honduras | 2016-05-03 |
| KY785472 | USA | 2016-09-20 |
| KY785473 | Dominican Republic | 2016-06-15 |
| KY785474 | USA | 2016-08-04 |
| KY785475 | Dominican Republic | 2016-04-07 |
| KY785476 | Dominican Republic | 2016-04-18 |
| KY785477 | Colombia | 2016-06-09 |
| KY785479 | Brazil | 2016-03-30 |
| KY785480 | Brazil | 2016-03-30 |
| KY785481 | Puerto Rico | 2016-04-12 |
| KY785484 | Dominican Republic | 2016-06-06 |
| KY817930 | Brazil | 2016-1 |
| KY989971 | Colombia | 2015-12 |
| MF073357 | Brazil | 2016-02-01 |
| MF073358 | Brazil | 2015-06-01 |
| MF073359 | Brazil | 2015-03-01 |
| MF159531 | USA | 2017-04-19 |
| MF352141 | Brazil | 2015-05-13 |
| MF384325 | Haiti | 2016-05-17 |
| MF434516 | Nicaragua | 2016-08-05 |
| MF434517 | Nicaragua | 2016-08-19 |
| MF434518 | Nicaragua | 2016-08-26 |
| MF434519 | Nicaragua | 2016-07-22 |
| MF434520 | Nicaragua | 2016-02-07 |
| MF434521 | Nicaragua | 2016-07-19 |
| MF434522 | Nicaragua | 2016-08-29 |
| MF438286 | Cuba | 2017-02-12 |
| MF574553 | Colombia | 2015-12 |
| MF574554 | Colombia | 2015-12 |
| MF574555 | Colombia | 2015-12 |
| MF574556 | Colombia | 2015-12 |
| MF574557 | Colombia | 2015-12 |
| MF574558 | Colombia | 2015-12 |
| MF574559 | Colombia | 2015-12 |
| MF574560 | Colombia | 2015-12 |
| MF574561 | Colombia | 2015-12 |
| MF574562 | Colombia | 2015-12 |
| MF574563 | Colombia | 2015-12 |
| MF574564 | Colombia | 2015-12 |
| MF574565 | Colombia | 2015-12 |
| MF574566 | Colombia | 2015-12 |
| MF574567 | Colombia | 2015-12 |
| MF574568 | Colombia | 2015-12 |
| MF574569 | Colombia | 2015-12 |
| MF574570 | Colombia | 2015-12 |
| MF574571 | Colombia | 2015-12 |
| MF574572 | Colombia | 2015-12 |
| MF574573 | Colombia | 2015-12 |
| MF574575 | Colombia | 2015-12 |
| MF574576 | Colombia | 2015-12 |
| MF574578 | Colombia | 2016-12 |
| MF574584 | Colombia | 2016-12 |
| MF574585 | Colombia | 2016-12 |
| MF574586 | Colombia | 2016-12 |
| MF574587 | Colombia | 2016-12 |
| MF783072 | Haiti | 2016-06-27 |
| MF794971 | Ecuador | 2016-05-01 |
| MF801377 | El Salvador | 2016-02-22 |
| MF801378 | Guatemala | 2016-07-21 |
| MF801380 | Guatemala | 2016-04-11 |
| MF801381 | Honduras | 2016 |
| MF801382 | Honduras | 2016 |
| MF801383 | Honduras | 2016-06-03 |
| MF801384 | Honduras | 2016-02-03 |
| MF801385 | Honduras | 2016-02-12 |
| MF801386 | Honduras | 2016-02-24 |
| MF801387 | Honduras | 2016-03-29 |
| MF801389 | Honduras | 2016-05-10 |
| MF801390 | Mexico | 2016 |
| MF801391 | Mexico | 2016-07-06 |
| MF801393 | Mexico | 2016-04-22 |
| MF801394 | Mexico | 2016-05-03 |
| MF801395 | Mexico | 2016-05-09 |
| MF801396 | Mexico | 2016-05-17 |
| MF801397 | Mexico | 2016-05-20 |
| MF801398 | Mexico | 2016-05-20 |
| MF801399 | Mexico | 2016-05-20 |
| MF801400 | Mexico | 2016-05-20 |
| MF801401 | Mexico | 2016-05-23 |
| MF801402 | Mexico | 2016-05-26 |
| MF801403 | Mexico | 2016-05-25 |
| MF801404 | Mexico | 2016-07-08 |
| MF801406 | Mexico | 2016-07-07 |
| MF801407 | Mexico | 2016-07-05 |
| MF801408 | Mexico | 2016-07-05 |
| MF801410 | Mexico | 2016-07-05 |
| MF801411 | Mexico | 2016-07-05 |
| MF801412 | Mexico | 2016-07-07 |
| MF801413 | Mexico | 2016-07-07 |
| MF801414 | Mexico | 2016-07-05 |
| MF801415 | Mexico | 2016-07-05 |
| MF801416 | Mexico | 2016-7 |
| MF801417 | Mexico | 2016-07-01 |
| MF801418 | Mexico | 2016-06-30 |
| MF801419 | Mexico | 2016-07-01 |
| MF801420 | Mexico | 2016-07-04 |
| MF801422 | Mexico | 2016-07-01 |
| MF801423 | Mexico | 2016-06-30 |
| MF801426 | Nicaragua | 2016-07-25 |
| MF988743 | USA | 2016-09-01 |
| MG494697 | Mexico | 2016-11-24 |
| MG770183 | Brazil | 2017-01-26 |
| MG770184 | Brazil | 2017-03-03 |
| MG770185 | Brazil | 2017-03-03 |
| MG770186 | Brazil | 2017-03-03 |
| MG827392 | French Polynesia | 2013-10-25 |
| MG976700 | French Polynesia | 2014-01-06 |
| MH063259 | Cuba | 2017-07-26 |
| MH063260 | Cuba | 2017-08-08 |
| MH063261 | Cuba | 2017-09-24 |
| MH063262 | Cuba | 2017-07-17 |
| MH063263 | Cuba | 2017-08-05 |
| MH063264 | Cuba | 2017-08-13 |
| MH063265 | Cuba | 2017-07-17 |
| MH157195 | Mexico | 2016-08-24 |
| MH157196 | Mexico | 2016-08-29 |
| MH157198 | Mexico | 2016-09-28 |
| MH157199 | Mexico | 2016-08-08 |
| MH157200 | Mexico | 2016-10-21 |
| MH157201 | Mexico | 2016-06-28 |
| MH157202 | Mexico | 2016-08-10 |
| MH157203 | Mexico | 2016-06-27 |
| MH157204 | Mexico | 2016-10-25 |
| MH157205 | Mexico | 2016-08-15 |
| MH157206 | Mexico | 2016-09-23 |
| MH157207 | Mexico | 2016-08-29 |
| MH157208 | Mexico | 2016-08-22 |
| MH157209 | Mexico | 2016-07-05 |
| MH157210 | Mexico | 2016-08-25 |
| MH157211 | Mexico | 2016-07-29 |
| MH157212 | Mexico | 2016-08-19 |
| MH157213 | Mexico | 2016-06-23 |
| MH157214 | Mexico | 2016-06-24 |
| MH179341 | Colombia | 2015-12-10 |
| MH513598 | Brazil | 2015-12-09 |
| MH513600 | Brazil | 2015-12-11 |
| MH544701 | Colombia | 2016-01-17 |
| MH882528 | Brazil | 2016-04-26 |
| MH882530 | Brazil | 2016-05-10 |
| MH882532 | Brazil | 2016-05-24 |
| MH882534 | Brazil | 2016-06-08 |
| MH882537 | Brazil | 2016-07-05 |
| MH882538 | Brazil | 2016-07-12 |
| MH882540 | Brazil | 2016-07-27 |
| MH882541 | Brazil | 2016-04-19 |
| MH882542 | Brazil | 2016-04-19 |
| MH882543 | Brazil | 2016-05-05 |
| MH882544 | Brazil | 2016-05-12 |
| MH882548 | Brazil | 2016-06-09 |
| MH882549 | Brazil | 2016-04-30 |
| MH900227 | Mexico | 2016 |
| MK049245 | Colombia | 2016-02-29 |
| MK049246 | Colombia | 2016-03-09 |
| MK049247 | Colombia | 2016-01-29 |
| MK049248 | Colombia | 2016-07-27 |
| MK049249 | Colombia | 2016-11-02 |
| MK049250 | Colombia | 2016-06-22 |
| MK049251 | Colombia | 2016-08-30 |
| MK049252 | Colombia | 2016-05-16 |
| MK560178 | Cuba | 2017-10-09 |
| MK560179 | Cuba | 2018-01-18 |
| MK713750 | Honduras | 2016-05-26 |
| NC035889 | Brazil | 2015 |
