# Supplementary material for: New Insights on the Zika Virus Arrival in the Americas and Spatiotemporal Reconstruction of the Epidemic Dynamics in Brazil
Source: Viruses. 2020 Dec 23;13(1):12. doi: 10.3390/v13010012 (PMC7824532; doi:10.3390/v13010012)
Supplement: Supplementary file 1 [file viruses-13-00012-s001.zip › Supplementary_Table_S2.docx]

Supplementary Table S2: Full-length ZIKV genomes and near-complete ZIKV genomes from Brazil (> 5000 nucleotides) available in GenBank up to January, 2019.

| **Accession Number** | **Country** | **Collection Date** |
| --- | --- | --- |
| KU321639 | SaoPaulo | 2015-3 |
| KU365777 | Para | 2015 |
| KU365778 | Para | 2015 |
| KU365779 | Para | 2015 |
| KU365780 | Paraiba | 2015 |
| KU497555 | Paraiba | 2015-11-30 |
| KU707826 | Bahia | 2015-07-01 |
| KU729217 | Ceara | 2015 |
| KU729218 | Para | 2015 |
| KU926309 | RiodeJaneiro | 2016-01-14 |
| KU926310 | RiodeJaneiro | 2016-01-29 |
| KU940224 | Bahia | 2015-08-01 |
| KU940227 | Bahia | 2015-07-15 |
| KU940228 | Bahia | 2015-07-01 |
| KX101060 | Bahia | 2015-5 |
| KX101061 | Bahia | 2015-5 |
| KX101064 | Bahia | 2015-4 |
| KX101066 | Bahia | 2015-5 |
| KX197192 | Pernambuco | 2015 |
| KX280026 | Paraiba | 2015 |
| KX520666 | Bahia | 2015-8 |
| KX811222 | Ceara | 2016-06-14 |
| KX830930 | RiodeJaneiro | 2016-03-01 |
| KX986760 | Pernambuco | 2016-2 |
| KY014296 | RiodeJaneiro | 2016-04-18 |
| KY014297 | RiodeJaneiro | 2016-04-12 |
| KY014301 | RiodeJaneiro | 2016-04-13 |
| KY014307 | RiodeJaneiro | 2016-03-28 |
| KY014308 | Bahia | 2016-03-23 |
| KY014309 | RiodeJaneiro | 2016-03-28 |
| KY014313 | RiodeJaneiro | 2016-04-05 |
| KY014317 | RiodeJaneiro | 2016-03-21 |
| KY014320 | RiodeJaneiro | 2016-03-23 |
| KY272991 | RiodeJaneiro | 2016-02-12 |
| KY441401 | SaoPaulo | 2016-02-29 |
| KY441402 | SaoPaulo | 2016-04-05 |
| KY441403 | SaoPaulo | 2016-01-11 |
| KY558989 | RioGrandedoNorte | 2015-02-23 |
| KY558990 | Pernambuco | 2016-01-15 |
| KY558991 | Pernambuco | 2016-01-19 |
| KY558992 | Pernambuco | 2016-01-06 |
| KY558993 | Pernambuco | 2016-01-18 |
| KY558994 | Pernambuco | 2016-01-18 |
| KY558995 | Pernambuco | 2015-05-13 |
| KY558996 | Pernambuco | 2015-05-13 |
| KY558997 | Pernambuco | 2015-05-14 |
| KY558998 | Pernambuco | 2015-06-15 |
| KY558999 | Pernambuco | 2016-07-10 |
| KY559000 | Pernambuco | 2015-08-09 |
| KY559001 | Alagoas | 2015-08-20 |
| KY559002 | Alagoas | 2015-09-09 |
| KY559003 | Alagoas | 2015-08-28 |
| KY559004 | Bahia | 2016-04-16 |
| KY559005 | SaoPaulo | 2016-04-18 |
| KY559006 | SaoPaulo | 2016-04-18 |
| KY559007 | SaoPaulo | 2016-04-18 |
| KY559008 | SaoPaulo | 2016-04-19 |
| KY559009 | SaoPaulo | 2016-04-19 |
| KY559010 | SaoPaulo | 2016-04-19 |
| KY559011 | SaoPaulo | 2016-04-19 |
| KY559012 | SaoPaulo | 2016-04-19 |
| KY559013 | SaoPaulo | 2016-04-24 |
| KY559014 | SaoPaulo | 2016-04-24 |
| KY559015 | SaoPaulo | 2016-04-24 |
| KY559016 | SaoPaulo | 2016-04-25 |
| KY559017 | SaoPaulo | 2016-05-19 |
| KY559018 | Tocantins | 2016-02-25 |
| KY559019 | Tocantins | 2016-05-24 |
| KY559020 | Tocantins | 2016-03-07 |
| KY559021 | Tocantins | 2016-03-10 |
| KY559022 | Tocantins | 2016-03-13 |
| KY559023 | Tocantins | 2016-03-22 |
| KY559024 | Tocantins | 2016-03-03 |
| KY559025 | RiodeJaneiro | 2016-01-15 |
| KY559026 | RiodeJaneiro | 2016-01-15 |
| KY559027 | RiodeJaneiro | 2016-02-16 |
| KY559029 | RiodeJaneiro | 2016-1 |
| KY559030 | RiodeJaneiro | 2016-1 |
| KY559031 | RiodeJaneiro | 2016-1 |
| KY559032 | RiodeJaneiro | 2016-1 |
| KY631492 | Amazonas | 2016-01-08 |
| KY785410 | RiodeJaneiro | 2016-03-27 |
| KY785426 | RiodeJaneiro | 2016-04-04 |
| KY785427 | RiodeJaneiro | 2016-03-30 |
| KY785429 | RiodeJaneiro | 2016-04-14 |
| KY785433 | RiodeJaneiro | 2016-04-08 |
| KY785437 | RiodeJaneiro | 2016-03-14 |
| KY785439 | RiodeJaneiro | 2016-04-08 |
| KY785446 | RiodeJaneiro | 2016-03-22 |
| KY785450 | RiodeJaneiro | 2016-04-12 |
| KY785455 | RiodeJaneiro | 2016-04-06 |
| KY785456 | RiodeJaneiro | 2016-04-15 |
| KY785479 | RiodeJaneiro | 2016-03-30 |
| KY785480 | RiodeJaneiro | 2016-03-30 |
| KY817930 | RiodeJaneiro | 2016-1 |
| MF073357 | NA | 2016-02-01 |
| MF073358 | NA | 2015-06-01 |
| MF073359 | NA | 2015-03-01 |
| MF352141 | Pernambuco | 2015-05-13 |
| MG770183 | SaoPaulo | 2017-01-26 |
| MG770184 | SaoPaulo | 2017-03-03 |
| MG770185 | SaoPaulo | 2017-03-03 |
| MG770186 | SaoPaulo | 2017-03-03 |
| MH513598 | MatoGrosso | 2015-12-09 |
| MH513600 | MatoGrosso | 2015-12-11 |
| MH882528 | SaoPaulo | 2016-04-26 |
| MH882530 | SaoPaulo | 2016-05-10 |
| MH882532 | SaoPaulo | 2016-05-24 |
| MH882534 | SaoPaulo | 2016-06-08 |
| MH882537 | SaoPaulo | 2016-07-05 |
| MH882538 | SaoPaulo | 2016-07-12 |
| MH882540 | SaoPaulo | 2016-07-27 |
| MH882541 | SaoPaulo | 2016-04-19 |
| MH882542 | SaoPaulo | 2016-04-19 |
| MH882543 | SaoPaulo | 2016-05-05 |
| MH882544 | SaoPaulo | 2016-05-12 |
| MH882548 | SaoPaulo | 2016-06-09 |
| MH882549 | SaoPaulo | 2016-04-30 |
| NC035889 | RioGrandedoNorte | 2015 |
