# Supplementary material for: New Insights on the Zika Virus Arrival in the Americas and Spatiotemporal Reconstruction of the Epidemic Dynamics in Brazil
Source: Viruses. 2020 Dec 23;13(1):12. doi: 10.3390/v13010012 (PMC7824532; doi:10.3390/v13010012)
Supplement: Supplementary file 1 [file viruses-13-00012-s001.zip › Supplementary_Table_S3.docx]

Supplementary Table 3: Median and 95% HPD of estimated introductions, as calculated by MCCTree, from Brazil to other American countries

| **Country** | **Year (95% HPD)** | **Bayes Factor (BF)** |
| --- | --- | --- |
| Colombia | April, 2015 (November, 2014 – June, 2015) | 1468.93 |
| Dominican Republic | June, 2015 (April, 2015 – July, 2015) | 87.79 |
| Haiti | June, 2014 (March, 2014 – September, 2014) | 131.71 |
| Honduras | October, 2014 (August, 2014 – December, 2014 | 100.5 |
| Puerto Rico | June, 2015 (April, 2015 – August, 2015) | 32.15 |
| Suriname | September, 2014 (June, 2014 – December, 2014) | 485.45 |
| Venezuela | June, 2015 (December, 2014 – October, 2015) | 3.39 |
