# Supplementary material for: New Insights on the Zika Virus Arrival in the Americas and Spatiotemporal Reconstruction of the Epidemic Dynamics in Brazil
Source: Viruses. 2020 Dec 23;13(1):12. doi: 10.3390/v13010012 (PMC7824532; doi:10.3390/v13010012)
Supplement: Supplementary file 1 [file viruses-13-00012-s001.zip › Supplementary_Table_S4.docx]

Supplementary Table 4: Median and and 95% HPD of estimated introductions, as calculated by MCC Tree, from Pernambuco to other Brazilian states

| **Brazilian state** | **Year (95% HPD)** | **Bayes Factor (BF)** |
| --- | --- | --- |
| Alagoas | April, 2014 (January, 2014 - July, 2014) | 76.59 |
| Bahia | June, 2014 (March, 2014 - August, 2014) | 15.34 |
| Mato Grosso | June, 2014 (March, 2014 – September, 2014) | 8.05 |
| Pará | February, 2014 (January, 2014 – July, 2014) | 21.6 |
| Paraíba | July, 2014 (April, 2014 – September, 2014) | 9.57 |
| Rio de Janeiro | July, 2014 (April, 2014 – August, 2014) | 831.6 |
| Rio Grande do Norte | May, 2014 (March, 2014 – August, 2014) | 37.06 |
| São Paulo | November, 2014 (August, 2014 - March, 2015) | 544.25 |
| Tocantins | September, 2014 (July, 2014 – January, 2015) | 3.57 |
